# Supplementary figures and images for: Evaluation of candidate reference genes for gene expression analysis in the brassica leaf beetle, Phaedon brassicae (Coleoptera: Chrysomelidae)
Source: PLoS One. 2021 Jun 3;16(6):e0251920. doi: 10.1371/journal.pone.0251920 (PMC8174695; doi:10.1371/journal.pone.0251920)

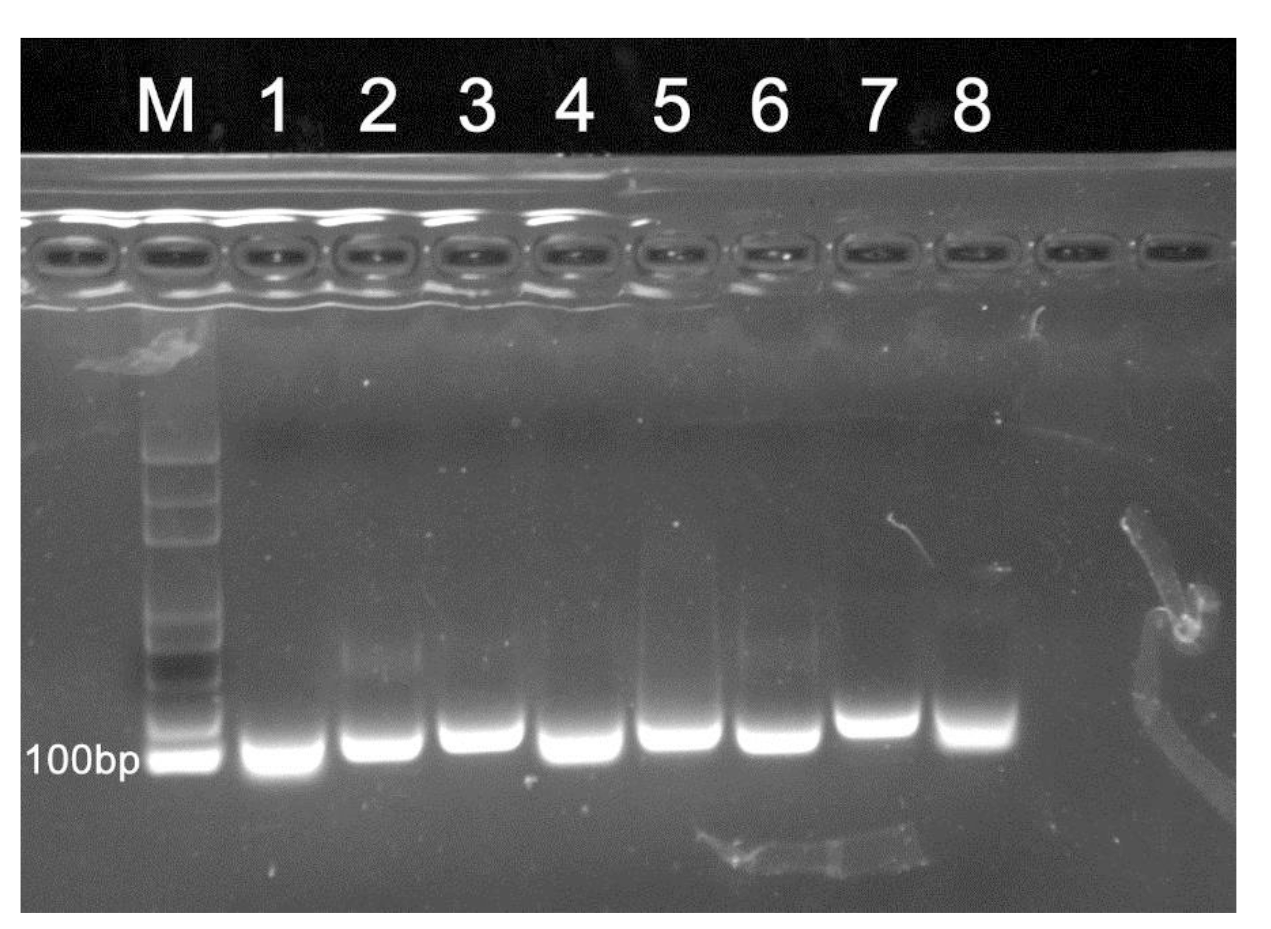

Supplement: S1 Fig — M, marker. Templates in the PCR reactions were as follows: 1) Actin2, 2) GAPDH, 3) RPL32, 4) α-TUB, 5) Actin1, 6) Ef-1α, 7) TBP, and 8) RPL19. (TIF) [file pone.0251920.s001.tif]

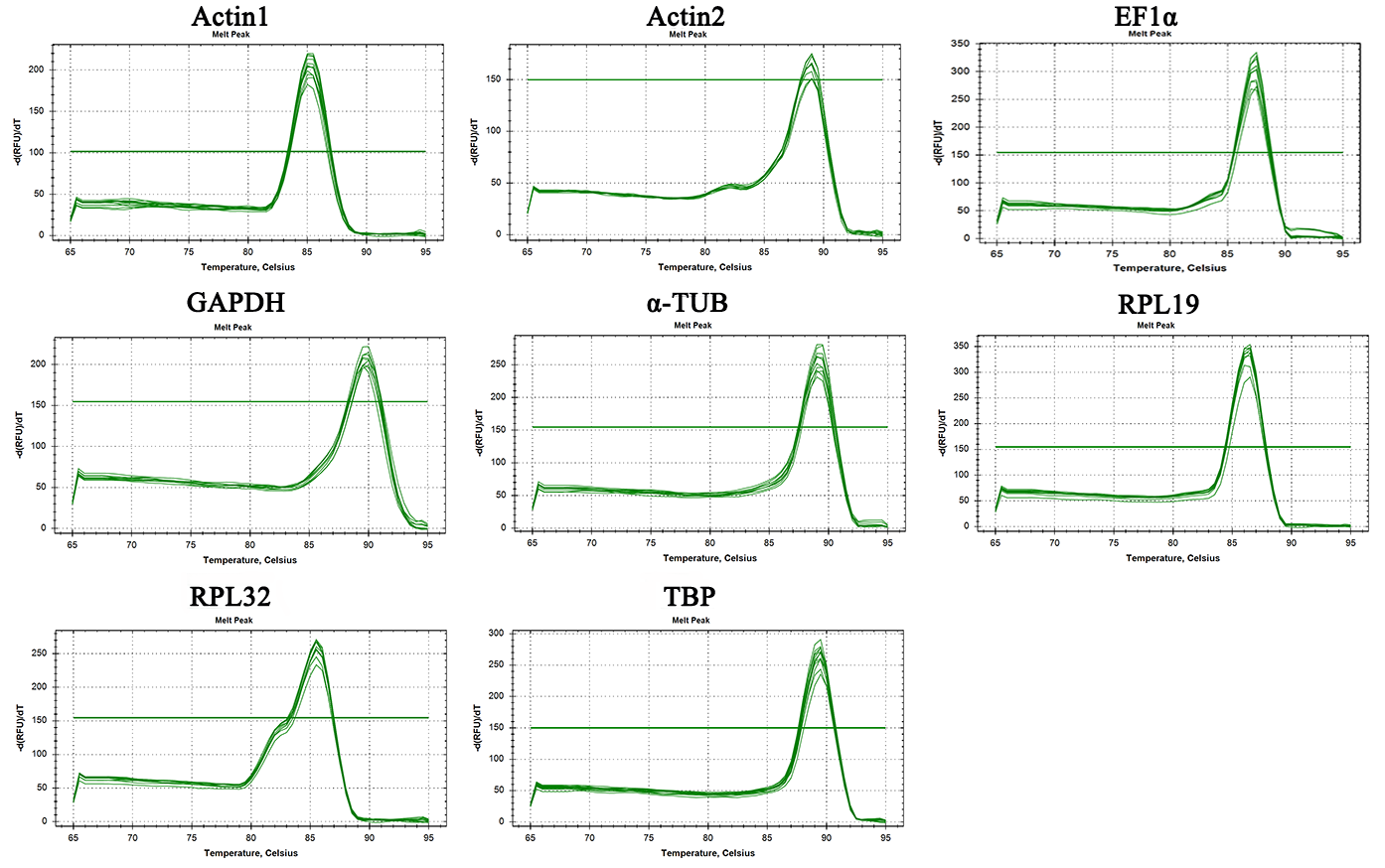

Supplement: S2 Fig — The gene-specific amplification was confirmed by a single peak in melting-curve analysis. (TIF) [file pone.0251920.s002.tif]

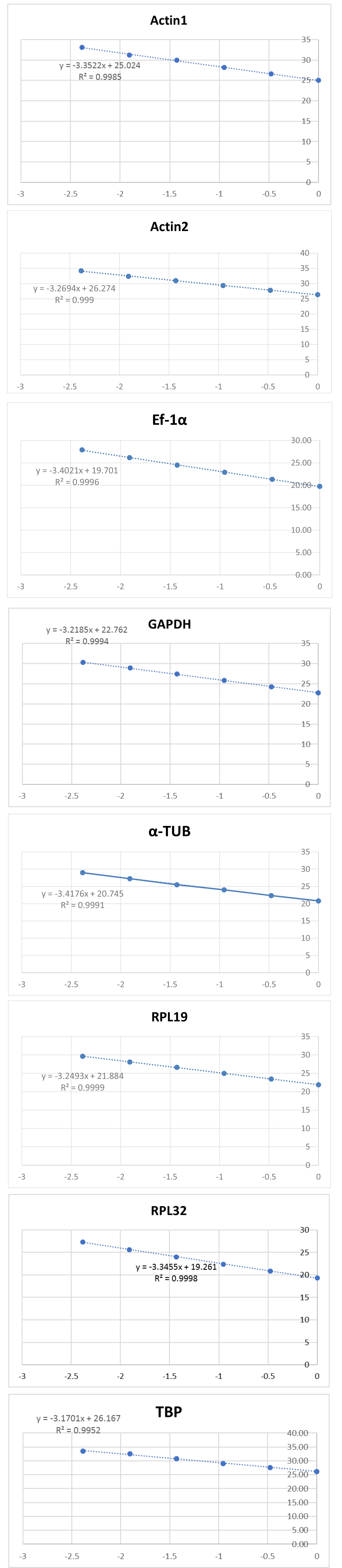

Supplement: S3 Fig — (TIF) [file pone.0251920.s003.tif]
