# Supplementary material for: Evaluation of candidate reference genes for gene expression analysis in the brassica leaf beetle, Phaedon brassicae (Coleoptera: Chrysomelidae)
Source: PLoS One. 2021 Jun 3;16(6):e0251920. doi: 10.1371/journal.pone.0251920 (PMC8174695; doi:10.1371/journal.pone.0251920)
Supplement: S1 Table — (DOCX) [file pone.0251920.s004.docx]

**S1 Table. Sequences of eight candidate reference genes.**

>Seq1 [Phaedon brassicae] Actin 1 MW509776

ATGGATGGGAAATTACCAGCTTGTGTTATTGATGTAGGAACTGGCTACACAAAATTAGGGTTCGCCGCGAATAAAGAGCCTCAGTTCATCATCCCTTCAGCCATCGCTATCAAAGAAACTGCAAAAGTAGGTGACACAACTTCTAGAAGATTAAATAAAGGTGTGGAAGATTTAGATTTTTTCATTGGGGATGAAGCATTTGATGCTACTGGATATGCAATAAAATATCCAGTAAGACATGGCCTTGTTGAAGACTGGGACCTGATGGAAAAATTCCTTGAACAATGCATTTTCAAATACCTACGTGCTGAGCCAGAAGATCACTATTTCCTCTTAACAGAACCTCCATTGAACACCCCTGAAAATAGAGAGTATATAGCAGAAATAATGTTTGAATCCTTCAATGTCCCAGGGCTGTATATAGCAGTGCAAGCAGTGCTAGCTCTAGCTGCAAGTTGGGCTAGTAGAAGTATAGAGGAAAGAACACTAACAGGGATTGTTATAGATAGTGGAGATGGTGTTACTCATGTTATTCCTGTAGCAGAAGGTTATGTCATTGGTAGTTGCATAAAGCACATTCCAATTGCTGGTCGAAACATAACCTATTTCATCCAGTCCCTTCTGAGAGAACGTGAAATCGGAATTCCACCAGAACAATCCCTAGAAACTGCTAAAGCCATTAAGGAAAAATACTGCTATATCTGTCCAGACATAGCTAAGGAATTCGCCAAGTACGACAGCGATCCGGGCAAGTGGATGAAAAGGTACGACGGCTCGAATTCGGTGACTAAACAACCGTTCGCCGTCGATGTCGGCTACGAGCGCTTCCTCGGCCCGGAGATATTCTTCCATCCGGAGTTCTCCAACCCGGACTTTACCATTCCGCTCTCGCAGATCGTCGATGACGTCATCCAGAATTGCCCGATCGACGTGAGGCGCCCTTTGTACGACAACATCGTCCTTTCGGGGGGCTCCACCATGTTCAAGGACTTCAGCAGACGGCTGCAGAGGGATATTAAAAGGACGGTGGACGCCAGGCTGAAGCTCAGCGAGACTTTGGGAGGGGGGCGGTTGAAGCCAAAACCCATTGATGTTCAAGTAGTGTCTCATCATATGCAAAGGTATGCAGTTTGGTTTGGAGGAAGTATGCTGGCGTCAACGCCTGAATTTTACACCGTCTGTCACACAAAGGAAGATTATGAAGAATATGGTCCTAGCATCTGCAGGCACAATCCAGTATTTGGAACAATGACGTAA

>Seq2 [Phaedon brassicae] Actin 2 MW509777

ATGGATAGTCAAGGCAGAAAAGTTATAGTTTGTGATAACGGTACAGGTTTTGTCAAATGCGGATATGCTGGGAGTAATTTTCCTGCTCACATATTCCCTTCGATGGTCGGAAGACCAATTATTAGAGCTGTGAACAAGATAGGAGATATCGAGGTTAAGGATTTGATGGTTGGAGATGAAGCTTCTGCTTTGAGATCACTTCTGGAAGTCAACTATCCCATGGAGAATGGCGTGGTTCGAAATTGGGAGGACATGTGCCATGTGTGGGACTATACTTTCGGTCCTCAAAAAATGGATTTGAATCCCAGTGAAACCAAAATATTGTTGACTGAACCACCGATGAATCCTACAAAAAATCGGGAGAAGATGATCGAAGTTATGTTTGAGAAGTATGGATTTGCGGGTGCTTATGTTGCTATTCAGGCTGTGTTGACACTGTATGCTCAGGGACTGTTGTCTGGTGTCGTAGTGGATTCGGGAGACGGGGTGACCCACATCTGTCCAGTGTACGAGGAGTACGCTCTGCCACACCTCACCAGGAGATTGGACATCGCCGGCAGAGACATCACCCGGTACCTCATCAAGTTACTCTTGCTCAGAGGGTACGCTTTCAACCATTCCGCTGATTTCGAGACTGTTCGTATGATGAAAGAGAAACTGTGCTACATCGGATACGACGTGGAGACAGAGCAGCGGTTGGCCTTGGAAACGACCATCCTGGTGGAGCCCTATACGCTGCCCGACGGGCGCGTAATCAAAGTGGGCGGGGAGAGGTTCGAGGCGCCCGAGGCGCTCTTCCAGCCGCATCTGATCAACGTCGAGGGCCAGGGCATCGCCGAGTTGGTCTTCAATACGATACAGGCCGCCGACATCGACATGCGCTCGGAGCTGTACAAGCACATCGTGCTGTCTGGCGGCTCGACGATGTACCCGGGCCTGCCTTCGAGGCTCGAGCGGGAAATCAAACAGCTCTACTTGGAGCGCGTGCTCAAAAACGACATCGAGAAGCTGAGCAAGTTCAAGATCCGGATCGAGGACCCGCCCAGGCGCAAGGACATGGTGTTCATAGGGGGCGCAGTATTGGCAGAAGTGATGAAGGATAGGGACGCCTTTTGGCTGTCAAAGCAAGAGTATGAAGAACAGGGGTTGAAGGTGCTGAAAAAGTTGGGCGCCAGGGTCGTTTGA

>Seq3 [Phaedon brassicae] alpha-tubulin MW509778

ATGCGTGAATGCATCTCCGTACACGTCGGCCAGGCCGGAGTCCAGATCGGCAACGCCTGCTGGGAGCTCTATTGCCTCGAGCACGGCATCCAGCCCGACGGACAGATGCCCTCCGACAAGACGGTGGGCGGCGGCGACGACAGCTTCAACACCTTCTTCAGCGAGACGGGGGCGGGCAAGCACGTACCGAGGGCGGTCTTCGTCGATTTGGAGCCGACGGTGGTAGACGAAGTGAGGACAGGGACCTACAGACAATTGTTCCATCCCGAACAGCTGATCACTGGCAAGGAAGATGCTGCTAACAACTATGCTAGGGGACACTACACCATTGGAAAGGAGATCGTGGATTTGGTTTTGGACAGGATCAGGAAGCTCGCTGACCAGTGCACTGGACTACAAGGTTTCCTGATCTTCCACTCGTTCGGCGGCGGTACCGGCTCCGGCTTCACCTCGCTGCTGATGGAGCGCCTCTCCGTCGACTACGGCAAGAAGTCGAAGCTCGAGTTCGCCATCTACCCGGCCCCGCAGGTGTCCACGGCCGTCGTGGAGCCCTACAACTCCATCCTGACCACGCACACCACTTTGGAGCATTCGGACTGCGCCTTCATGGTCGACAACGAGGCTATTTACGATATTTGCAGGAGGAATTTGGACATCGAGAGGCCCACTTACACCAACTTGAACAGGCTTATTGGACAAATCGTGTCTTCCATCACTGCTTCTCTGCGTTTCGACGGAGCCCTCAACGTGGACCTCACCGAGTTCCAGACCAACTTGGTGCCGTACCCCCGTATCCACTTCCCGCTGGTGACTTACGCCCCCGTCATTTCTGCCGAAAAGGCGTATCACGAGCAACTTTCGGTGGCCGAGATCACCAACGCCTGCTTCGAGCCGGCCAATCAGATGGTGAAATGCGACCCGAGACACGGCAAGTACATGGCTTGCTGCATGTTGTACAGGGGGGACGTGGTGCCCAAGGACGTGAACGCGGCCATCGCTACGATCAAGACCAAGAGGACCATTCAGTTTGTGGACTGGTGTCCCACCGGATTCAAGGTCGGTATCAACTACCAACCACCCACCGTGGTGCCCGGCGGCGATCTCGCTAAAGTCCAGCGCGCCGTCTGCATGTTGTCCAACACCACCGCCATCGCCGAGGCTTGGGCGCGCCTCGACCACAAGTTCGACCTCATGTACGCCAAGAGGGCGTTCGTGCACTGGTACGTCGGTGAGGGCATGGAGGAGGGCGAGTTCTCCGAGGCCAGAGAGGATTTGGCCGCTCTCGAGAAGGATTACGAAGAGGTCGGCATGGATTCCGGAGAAGGCGAAGGAGAGGGAGCTGAAGAATACTAG

>Seq4 [Phaedon brassicae] elongation factor 1 alpha MW509779

ATGGGTAAAGAAAAGGTTCACATTAACATTGTCGTCATCGGTCACGTAGATTCTGGTAAATCTACTACTACTGGACACTTGATCTACAAATGTGGTGGTATTGACAAACGTACCATCGAAAAATTCGAGAAGGAAGCCCAGGAAATGGGAAAAGGTTCCTTCAAGTATGCATGGGTACTTGACAAACTGAAGGCTGAACGTGAACGTGGTATCACCATCGATATTGCCCTATGGAAGTTTGAAACTGCCAAGTACTATGTTACTATCATTGATGCCCCTGGACACAGAGATTTCATCAAGAACATGATCACTGGTACATCGCAGGCCGATTGTGCTGTACTTATTGTTGCTGCTGGTACTGGTGAATTCGAAGCTGGTATCTCCAAAGACGGACAGACCCGTGAGCATGCTCTTCTTGCTTTCACCCTTGGAGTAAAACAACTTATTGTCGGTGTCAACAAAATGGACTCCACTGAACCACCATACAGTGAATCCCGTTTTGAGGAAATTAAGAAGGAAGTATCCTCTTACATCAAGAAGATCGGTTACAACCCAGCTGCTGTTGCTTTTGTGCCAATTTCCGGATGGCATGGAGACAACATGTTGGAATCATCTGCCAAGATGCCATGGTTCAAGGGATGGGCTGTTGAACGTAAAGAAGGAAAGGCCAATGGTAACTGCTTGATTGAAGCTCTAGATGCCATCCTCCCGCCTTCCCGTCCAACTGAGAAGCCTCTGCGTCTTCCACTCCAGGATGTCTACAAAATTGGAGGTATTGGAACAGTACCAGTAGGTCGTGTGGAAACTGGTGTGCTGAAGCCCGGTATGGTTGTGGTCTTCGCCCCAGCCAACATCACCACTGAAGTGAAATCCGTGGAGATGCACCACGAAGCTCTCCAGGAAGCCGTACCCGGAGACAACGTCGGTTTCAACGTCAAGAACGTCTCCGTCAAGGAATTGCGTCGTGGGTACGTAGCCGGAGACACCAAAGCCAGCCCTCCCAAGGGGGCCACCGACTTCAACGCCCAAGTCATCGTCCTGAACCACCCTGGTCAGATCTCCAACGGATACACGCCTGTGTTGGATTGTCACACTGCCCACATTGCCTGCAAATTCGCTGAAATCAAGGAGAAGGTCGACCGTCGATCGGGAAAGACGACTGAAGAAAACCCCAAAGCCATCAAGTCCGGAGACGCCGCCATTGTCAACTTGGTCCCCACTAAGCCAATGTGTGTGGAATCCTTCCAGGAGTTCCCCCCTCTGGGAAGGTTCGCTGTCCGTGACATGAGGCAAACCGTTGCCGTAGGAGTCATCAAGAGTGTCAACTTCAAGGACCCCACTGCCGGAAAGGTCACAAAAGCTGCCGAGAAAGCACAGAAGAAGAAATAG

>Seq5 [Phaedon brassicae] glyceraldehyde-3-phosphate dehydrogenase MW509780

ATGGTGAAAATGGGAATAAACGGTTTCGGGCGCATCGGGCGTCTGGTGCTGCGCGCAGCTCTCGAGCGCGGCGTGGAGGTGGTGGCCGTCAACGACCCCTTCCTCAACGTCGAGTACATGGTGTACCTGTTCAAGTACGACTCGACGCACGGGCGGTACAAGGGCTGCGTGGCCGCCGACGGCAACAACCTGGTCGTCAACGGGGTCAAGATCGCCGTGTACTCCGAGAAGGACCCTAAGCAAATCCCATGGGGTAAACATGGTGCCGACTACGTAGTCGAATCCACCGGTGTATTCACCACCATTGAGAAGGCTTCCGCCCATTTGGATGGAGGCGCCAAGAAAGTCATCATTTCTGCCCCATCTGCTGACGCTCCAATGTATGTCGTCGGTGTCAATCTTGATGCTTACAACCCATCTGATAAGGTAATCTCGAACGCCTCGTGCACCACCAACTGCCTGGCCCCGCTCGCCAAGGTCATCCACGACAACTTCGAGATCGTCGAAGGCCTGATGACGACCGTCCATGCGACCACCGCCACCCAGAAGACTGTCGACGGTCCTTCGGGCAAGCTGTGGCGCGACGGGCGCGGCGCCGGCCAGAACATCATCCCGGCGTCGACGGGCGCCGCCAAGGCCGTCACCAAGGTCATCCCCTCTCTTGTCGGCAAACTCACCGGTATGGCCTTCCGTGTGCCGGTCGCCAACGTGTCCGTGGTTGACTTGACCGTCCGCCTGGGCAAACCGGCCTCTTACGACGAGATCAAAAACAAGATCAAGGAGGCTTCGGAGGGCCCTCTGAAGGGTATCCTCGGCTACACCGAGGACGCCGTCGTCTCGTCCGATTTCATCGGGGACACCCATTCTTCCGTCTTCGATGCCACCGCCGGCATCCAACTCAACCCGGGATTCGTCAAACTTATTTCGTGGTACGACAACGAGTACGGGTACTCGAGCAGGGTCATTGACCTGATCAAGTTCTGCGCCACCAAAGATGCTTAA

>Seq6 [Phaedon brassicae] ribosomal protein L19 MW509781

ATGAGTTCCTTGAAACTTCAAAAGAGGCTAGCAGCCTCGGTAATGCGATGTGGTAAGAAGAAAGTATGGTTGGATCCAAATGAAATCAACGAAATCGCCAACACAAACTCAAGACAAAACATCCGTAAGCTGATCAAGGATGGTCTCATTATCAAGAAACCAGTGGCAGTACATTCAAGAGCCCGTGTACGCAAGAACACTGAAGCCAGAAGGAAGGGAAGGCATTGTGGGTTTGGAAAGAGGAAGGGTACGGCGAATGCCCGTATGCCTCAAAAGGAACTGTGGGTACAGCGCATGCGCGTGCTCAGACGCCTTCTTAAAAAATACCGCGAGGCCAAAAAGATCGACCGCCATCTTTACCACGCCCTGTACATGAAGGCGAAGGGTAACGTGTTCAGGAACAAGCGTGTCCTCATGGAGTACATCCACAAGAAGAAGGCTGAGAAGGCCAGGGCTAAGATGCTGTCTGACCAGGCGAACGCCAGGAGGTTGAAGGTGAAACAGGCCAGGGAGCGCAGGGAAGAAAGGATCGCTACGAAGAAACAGGAAGTCCTGGCTAATTACCAGAGGGAGGACGAGGCTGCTGCAGCCAAAAAAATAGTGTTTTTTAAGATGTGA

>Seq7 [Phaedon brassicae] ribosomal protein L32 MW509782

ATGGCCATCAGACCTGTTTACAGGCCTGATATAATAAAAAAGAGGACAAAAAAGTTCATCAGACATCAGTCTGATAGGTATGGTAAACTAAAGCGAAACTGGCGTAAACCGAAAGGTATTGACAACAGAGTGAGAAGGCGTTTCAAGGGTCAGTTTTTGATGCCAAATATTGGTTATGGATCAAATGCAAAAACTCGTCATATGTTACCTACTGGATTCCGTAAAGTTTTAGTACACAACGTTAGGGAACTCGAAGTCCTTCTCATGCAGAACCGCAAATACTGCGCTGAAATCGCGCACGGGGTGTCGTCGAAGAAGCGCAAGGAGATCGTCGAGAGGGCGCAGCAGTTGAGCATCAGGGTTACCAACGGCCACGCACGACTACGTAGCCAAGAAAACGAATAA

>Seq8 [Phaedon brassicae] TATA box-binding protein-like MW509783

ATGGCTACTGTTGTTCAGAAAAATGGTATTAAGCCACTATCTAATGGAAATAGTTTGGTGCATCATCAGATGCAGAATATCAATGGTTATGAACATGTTGAAGTGTTGAATGACAATACAGAGTGTGATTCTCAACAAAACAATGAGCAAAATGATTCTTGTGAACCTGATAGTGAACAGACTGAAATTGAGCAGATTATTGAAGAACCAGAAATAGACATTGTCATTAACAATGTAGTTTGCAGTTTTAGTGTAAGGTGTCATTTAAATTTACGTGAAATCGCACTATCTGGTACAAATGTTGAATATCGTAAAGAAAATGGCATGGTTACAATGAAACTACGAAGACCTTATACAACAGCAAGTATTTGGTCATCAGGAAAAATCACCTGTACAGGTGCTACTAGTGAAGATGCAGCTAAGCAAGCAGCAAGAAGGTTTGCACGCTGCTTACAGAAATTGGGGTTCAACACTAGATTCAACAATTATAGAGTGGTTAATGTACTTGGAACATGCTCGATGCCCTTCTCAATAAGAATCAGTTCCTTTTCTGCACGACATAGAGAAGCAGATTATGAACCAGAGTTACATCCAGGGGTCACATATAAGCTGAAAAGTCCAAAAGCCACCCTGAAAATATTTTCAACAGGCAGTGTCACAGTTACTGCGCCTAGTGTAGCTGATGTACAAGCTGCCATCGAATACATATTTCCGTTAGTCTACGAATTCAGAAAAGAGAGAACCAAAGAAGAAAAAGAGGCATTAGCAAAAAAGAAACTGAAGCAGTATGGAGAAATTCCTTTGGAACATGAAGAAGCAGATGAAGCAATGAGTGAATCTGAAAATGTTGAAGAATCATGGGACTGA
