# Supplementary material for: Evaluation of candidate reference genes for gene expression analysis in the brassica leaf beetle, Phaedon brassicae (Coleoptera: Chrysomelidae)
Source: PLoS One. 2021 Jun 3;16(6):e0251920. doi: 10.1371/journal.pone.0251920 (PMC8174695; doi:10.1371/journal.pone.0251920)

The agarose gel electrophoresis of eight candidate reference genes. M, marker; Templates in the PCR reactions were as follows: 1) Actin2; 2) GAPDH; 3) RPL32; 4) α-TUB; 5) Actin1; 6) Ef-1α; 7) TBP; and 8) RPL19.

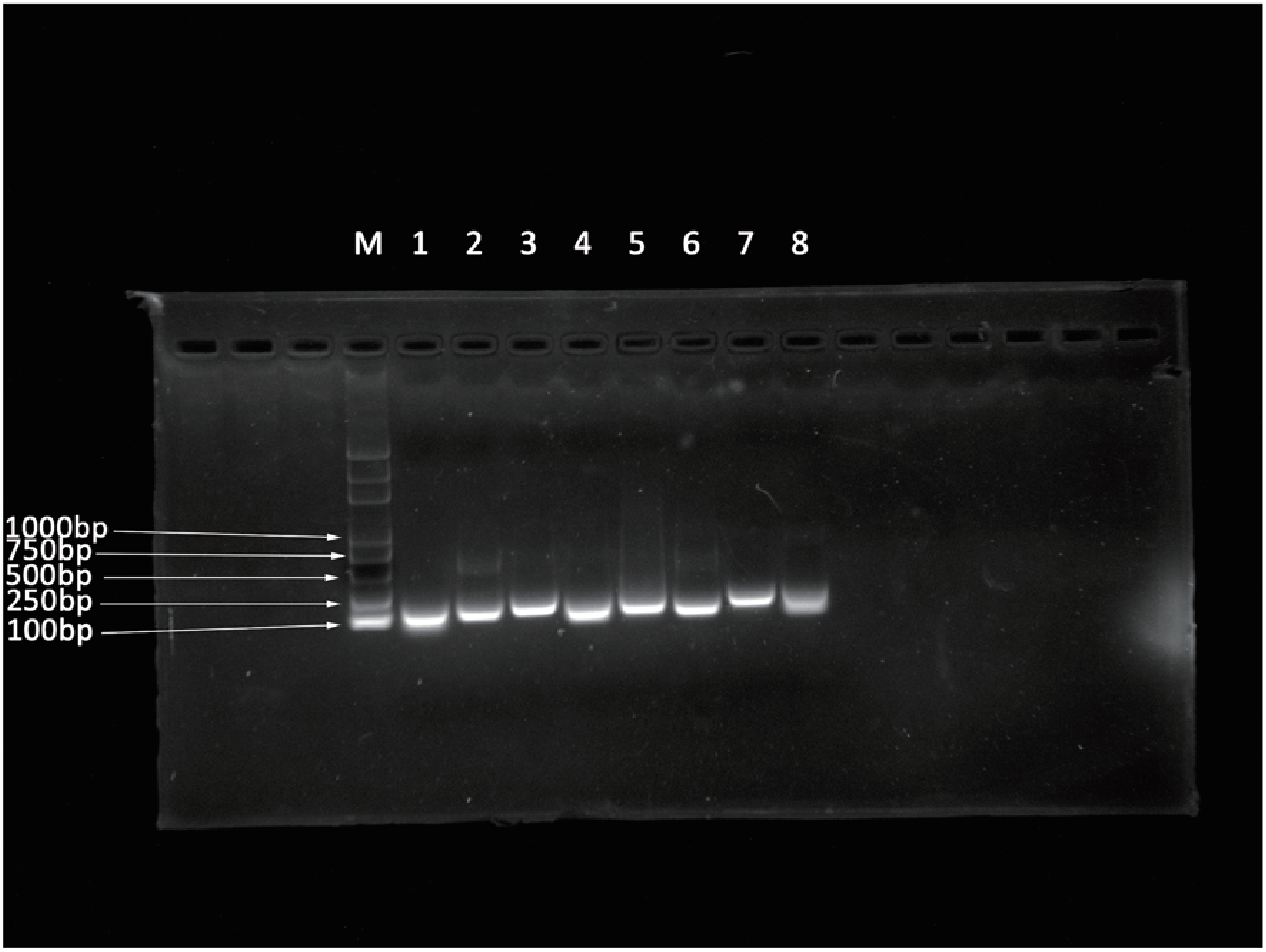

Supplement: S1 Raw images — (PDF) [file pone.0251920.s005.pdf]
