# Supplementary material for: Evaluation of candidate reference genes for gene expression analysis in the brassica leaf beetle, Phaedon brassicae (Coleoptera: Chrysomelidae)
Source: PLoS One. 2021 Jun 3;16(6):e0251920. doi: 10.1371/journal.pone.0251920 (PMC8174695; doi:10.1371/journal.pone.0251920)
Supplement: Attachment — Submitted filename: response.pdf [file pone.0251920.s006.pdf]

Dear editor and reviewers,

On behalf of my co-authors, we are grateful for giving us an opportunity to revise our manuscript, and we appreciate the anonymous reviewers for their positive and constructive comments on our manuscript. Based on these comments, we tried our best to polish the manuscript. The manuscript was modified systemically using the track change mode. The corrections are listed below point by point.

Review Comments to the Author

Reviewer #1: Ma et al. 2020 reports the validation of several housekeeping genes under different conditions in an important vegetable pest. This is one of several studies that document the importance of validating reference genes in different conditions, in order to obtain a set of standard genes that work best for appropriate conditions. The ms is well written, and analyses seem appropriate.

I have just a few comments below.

line 57: 'remains' is misspelled

Respond: Thanks for your correction.

line 97: I recommend to mention in this paragraph the 8 genes used in the study

Respond: Thanks for your suggestion.

line 115: …25°C ± 1, …

Respond: Thanks for your suggestion. We checked the same usage in reference to Chen et al. (2017), Ozaki et al. (2011), and etc.

1. Ozaki K, et al. A gustatory receptor involved in host plant recognition for oviposition of a swallowtail butterfly. Nat Commun. 2011. doi: 10.1038/ncomms1548.
2. Chen C, et al. Protein kinase C delta phosphorylates ecdysone receptor B1 to promote gene expression and apoptosis under 20-hydroxyecdysone regulation. Proc Natl Acad Sci USA. 2017.114:7121-7130.

line 116: ..70% ± 10,…

line 126-129: how were the insects exposed, by feeding, filter paper petri dish, injection? Please clarify.

Respond: Thanks for your suggestion. In this work, the insecticide exposure experiment was performed following a leaf-dipping method (Cui et al., 2018; Lutz et al., 2018). In the present version, we make a detailed description as follows, 'the leaf discs of radish (2 cm diameter) were dipped in the solutions with insecticide for 10 s, then left to air-dry at room temperature. Each piece of the dipped leaf discs was placed in a Petri dish, into which four larvae were transferred'.

1. Cui L, et al. Resistance selection of indoxacarb in *Helicoverpa armigera* (Hübner) (Lepidoptera: Noctuidae): Cross-resistance, biochemical mechanisms and associated fitness costs. Pest Manag Sci. 2018, 74:2636-2644.
2. Lutz A, et al, Lethal and sublethal effects of chlorantraniliprole on *Spodoptera cosmioides* (Lepidoptera: Noctuidae). Pest Manag Sci. 2018, 74:2817-2821.

line 131: …2h and 4h…

Respond: Thanks for your correction.

line 131: …It was worth emphasizing that… this sentence is not needed, stat at ..Each experiment contained….

Respond: Thanks for your suggestion. This sentence was modified as you suggested.

line 141: 'specimen' is misspelled

Respond: Thanks for your correction.

line 159: volume

Respond: Thanks for your correction.

line 214: 'with' regard is misspelled

Respond: Thanks for your correction. In the present version, this was corrected to 'in regards to'.

line 290: Ribosomal proteins are known….

Respond: Thanks for your correction.

line 290-310: other example of chrysomelid species, *Diabrotica undecimpunctata* howardi, which is an important maize pest in North America, could be included in the discussion; RPS9 and EF-1α were documented each to be the most stable genes in one of the different conditions (Basu et al. 2019, https://doi.org/10.1038/s41598-019-47020-y).

Respond: Thanks for your constructive suggestion. In the current version, more references, including Basu et al., (2019), were employed to consolidate our arguments, and we rewrote the last paragraph to deepen our discussions, according to your comments. Additionally, more assumptions and summary were made in the end of each paragraph.

line 335: delete 'that'

Respond: Thanks for your correction.

line 340-345: I recommend to mention the best reference genes for each experimental conditions here.

Respond: Thanks for your suggestion. In the section of Results, the best reference genes were recommended for each experimental conditions as follows, 'Integrating the evaluation of all four programs, the comprehensive ranking of candidate reference genes was determined by RefFinder (Table 2), wherein *RPL19* and *TBP* were considered as the best reference genes across different developmental stages, *RPL32* and *Ef-1α* were recommended for tissue expression analysis, *RPL32* and *TBP* were identified as the optimal candidates for the temperature experiment, and *RPL32* and *RPL19* were selected for the insecticide treatment'.

Reviewer #2: Authors Ma et al. performed a study to evaluate candidate reference genes for molecular studies in Brassica Leaf Beetle, a serious pest of brassicaceous vegetables in East and South Asia. The research is important as identifying the most stable reference genes for a species is required for many molecular research based on the quantitative real-time PCR technique. In general, the experimental design and data analysis of this study are scientifically sound. However, the clarification of data presentation, language quality, discussion and citation need to be enhanced significantly. Following are some of my specific suggestions.

1. The English of this manuscript should be polished considerably. There are many errors (spelling, grammar, etc.) and clarification problems. Authors should rewrite some sentences to make it clear. Honestly speaking, asking for editorial help from a native speaker will be optimal.

Respond: Thanks for your constructive suggestion, and we check our writing thoroughly to prevent the misleading expression.

1.1. Title: Evaluation, not Evalution

Respond: Thanks for your correction.

1.2. Title: change "in a Brassica Leaf Beetle" to "in the Brassica Leaf Beetle"

Respond: Thanks for your correction.

1.3. Line 55: change "insecticide" to "insecticides"

Respond: Thanks for your suggestion.

1.4. Rewrite the sentence in Lines 54-57. Two sentences instead of one will be ideal. Using However instead of but

Respond: Thanks for your suggestion. In the current version, we cited more references to describe the field management against *P. brassicae* as follows, 'however, the application of insecticides was not always effective against *P. brassicae* due to its complex lift history and high fecundity (Wang et al., 2007), and previous studies reported the exploration of a cadherin-based peptide as an enhancer for Cry3Aa-based products in controlling *P. brassicae* (Gao et al., 2011)'. This sentence was removed to make a coherent expression of the issue.

1. Wang X, Xue F, Tan Y, Lei C. The role of temperature and photoperiod in diapause induction in the brassica leaf beetle, *Phaedon brassicae* (Coleoptera: Chrysomelidae). European Journal of Entomology, 2007, 104(4):693-697.
2. Gao Y, Jurat-Fuentes JL, Oppert B, Fabrick JA, Liu C, Gao J, Lei Z. Increased toxicity of Bacillus thuringiensis Cry3Aa against *Crioceris quatuordecimpunctata*, *Phaedon brassicae* and *Colaphellus bowringi* by a Tenebrio molitor cadherin fragment. Pest Management Science. 2011, 67(9):1076-81.

1.5. Line 57: change “remians ambiguous” to “remain unknown”.

Respond: Thanks for your correction.

1.6. Line 57-58: rewrite this sentence. It is not clear! You did not mention anything of the functional gene research in your previous sentences.

Respond: Thanks for your suggestion. In the current version, we rewrote the sentence as follows, ‘To identify novel target genes for controlling *P. brassicae*, accurate quantification of gene expression under different conditions is indispensable’.

1.7. Line 74: change “between” to “among”

Respond: Thanks for your correction.

1.8. Line 82: change “treatments” to “abiotic conditions”

Respond: Thanks for your suggestion.

1.9. Lines 87-91: using at least two sentences here instead of only one sentence

Respond: Thanks for your suggestion.

1.10. Line 119: low-case the subtitle except the first letter in order to keep consistence with other subtitles “Experimental Treatment and Sample collection”

Respond: Thanks for your correction.

1.11. Line 214: change “Wih” to “With”

Respond: Thanks for your correction. In the present version, this was corrected to ‘in regards to’.

1.12. Line 221: change “besides” to “and”

Respond: Thanks for your suggestion.

1.13. Line 230: add “ones” after “RPL19 as the most stable”

Respond: Thanks for your suggestion.

1.14. Lines 225-226: change this sentence to “ranked RPL32, TBP and α-TUB as the top three reference genes”

Respond: Thanks for your correction. In the present version, this sentence was corrected to ‘NormFinder and geNorm ranked *RPL32*, *TBP* and *α-TUB* as the top three reference genes’.

1.15. Line 227: add “the” – in “the” pesticide exposure experiment

Respond: Thanks for your correction.

2. Material and method

2.1. Line 111- please provide the background of pesticide usage of this insect population. Is there any pesticide application before and after keeping in the laboratory condition?

Respond: Thanks for your suggestion. In the section of ‘Insect Rearing’, we made a description as follows, ‘This strain has been reared for six generations without exposure to chemical insecticides’.

2.2. Line 143- please provide the accession numbers of these candidate reference genes in NCBI database

Respond: Thanks for your suggestion. In the current version, the sequences and accession numbers of the candidate reference genes have been supplemented in Table 1 and Table S1.

2.3. Line 146: any accession codes available for the RNA-seq transcriptome database of Phaedon brassicae? Or published papers? If yes, please cite them or provide this information

Respond: Thanks for your suggestion.

2.4. Line 165: Besides four individual programs you listed here, which program was used for ranking these candidate genes in general? (Table 2) RefFinder program? Please find and cite these references: Xie et al. Plant Molecular Biology 2012; Morales et al. International Journal of Biological Sciences 2016

Respond: Thanks for your suggestion. In the end of this paragraph, we described how the comprehensive ranking of candidate reference genes was determined by RefFinder as follows, "Finally, a comprehensive analysis tool RefFinder (https://www.heartcure.com.au/reffinder/#) was used to integrate results of the four different analytical methods and assess the rank of reference genes based on their geometric mean". Furthermore, these references were cited as suggested.

2.5. Line 181: Please provide the accession number for sHSP20.0

Respond: Thanks for your suggestion. The accession number was supplemented in the current version.

3. Results:

3.1. Table 1. Please provide the accession numbers for these candidate genes

Respond: Thanks for your suggestion. In the current version, the accession numbers were supplemented in Table 1, and meanwhile the sequences of the eight candidate reference genes were added in supplementary data (Table S1).

3.2. Figure 1: should be moved to supplementary data

Respond: Thanks for your suggestion. In the current version, Figure 1 was transferred to Supplementary data.

3.3. Table 2: shows a "recommendation" of the most stable genes. However, in the M&M authors failed to provide the program used to recommend. Please reference previous published papers such as Xie et al. 2012; Morales et al. 2016, add more details in M&M and References

Respond: Thanks for your suggestion. In the section of Materials and methods, we described how the comprehensive ranking of candidate reference genes was determined by RefFinder as follows, "Finally, a comprehensive analysis tool RefFinder (https://www.heartcure.com.au/reffinder/#) was used to integrate results of the four different analytical methods and assess the rank of reference genes based on their geometric mean".

3.4. Figure 2: keep either plural or single consistent. For example, developmental stages, tissues, temperature conditions…

Respond: Thanks for your suggestion. In the current version, these items in Figure 2 have been modified.

4. Citation and Introduction/Discussion: There are many similar types of research published during past decade. However, authors only cited a few of them.

Respond: Thanks for your suggestion. In the current version, more recent reference articles were attached to consolidate our arguments.

When author gave the Introduction, only 3 Phaedon brassicae papers were cited in the first paragraph (Lines 44-58)!! Some physiology background of this species is required in guiding authors to choose developmental stages, tissues, thermal stresses and insecticide stresses for their study.

Respond: Thanks for your suggestion. In the current version, more references were employed to elucidate the physiology background of *Phaedon brassicae* as follows, "this beetle escapes from predators by dropping from host plants and interestingly their larvae exhibit less frequently dropping behavior than adults in response to attacks (Matsubara et al., 2018). In the Yangtze River Valley, there are two distinct infestation peaks in the field: the single spring generation and the two generations in autumn, which undergo aestivating and hibernating imaginal diapause in soil, separately (Wang et al., 2007a, 2007b; Wang et al., 2009). This beetle is a typical short-day species in which low temperature would enhance the induction of its winter diapause while high temperature suppresses the incidence of its summer diapause (Wang et al., 2007)".

1. Gao Y, Jurat-Fuentes JL, Oppert B, Fabrick JA, Liu C, Gao J, Lei Z. Increased toxicity of Bacillus thuringiensis Cry3Aa against Crioceris quatuordecimpunctata, *Phaedon brassicae* and *Colaphellus bowringi* by a Tenebrio molitor cadherin fragment. Pest Management Science. 2011, 67: 1076-81.
2. Matsubara S, Sugiura S. Host plant architecture afects the costs of dropping behaviour in *Phaedon brassicae* (Coleoptera: Chrysomelidae). Applied Entomology and Zoology. 2018, 53: 501-508.
3. Wang X, Xue F, Tan Y, Lei C. The role of temperature and photoperiod in diapause induction in the brassica leaf beetle, *Phaedon brassicae* (Coleoptera: Chrysomelidae). European Journal of Entomology, 2007a, 104: 693-697.
4. Wang X, Zhou X, Lei C. Development, survival and reproduction of the Brassica leaf beetle, Phaedon brassicae Baly (Coleoptera: Chrysomelidae) under different thermal conditions. Pan-Pacific Entomologist. 2007b, 83: 143-151.
5. Wang X, Zhou X, Lei C. Adaptation to aging and absence of host-plant in the brassica leaf beetle, Phaedon brassicae. Chinese Bulletin of Entomology 2009, 46: 403-407.

Similarly, in the Discussion section, please cite more papers, especially some review or representative ones (e.g. insect pests, beneficial insects, mites, other arthropods or animals, plants, etc.) that have

summarized the key questions in this research topic. Otherwise, this research is just simply mimicking previous publications in a different species!

Respond: Thanks for your constructive suggestion. In the current version, more references were employed to consolidate our arguments, and we rewrote the last paragraph to deepen our discussion, according to your comments.

Reviewer #3: The authors evaluated the potential of 8 genes as the reference gene that could be used in RT-qPCR assay. Four algorithms were applied to score each candidate gene across four types of samples and yielded 4 suites of optimal reference genes combination for each of the detected 4 given type of samples. The experiments were carefully designed and the results are reliable. This work is important to support the future gene functional study of Phaedon brassicae. The ms was carefully written with a clear logic flow. However, I would advise authors to get this manuscript checked by native English speaker. I believe this manuscript is a good piece of work but also has lot of scope for English language corrections. No major problem could be found. But some minor revisions need to be made before publication.

1. Why do you select the doses of acetamiprid at 2 μg/ml, dinotefuran at 5 μg/ml, and abamectin at 0.18 μg/ml? How did the authors obtain the sublethal dose of different pesticides? Why use PBS as control?

Respond: In our preliminary experiment, bioassays were conducted with the second-instar larvae following a leaf-dipping method, and the toxicity of acetamiprid, dinotefuran, and abamectin to the second-instar larvae were determined. In brief, the leaf discs of radish (2 cm diameter) were dipped in the solutions with insecticide for 10 s, then left to air-dry at room temperature. Each piece of the dipped leaf discs was placed in a Petri dish, into which four larvae was transferred. In the insecticide exposure experiment, the concentration of LC_{10} was selected as the sublethal dose.

2. The discussion section need more concise.

Respond: Thanks for your constructive suggestion. In the current version, more references were employed to consolidate our arguments, and we rewrote the last paragraph to deepen our discussion, according to your comments.

Specific points:

1. Please add the version and literature for each software.

Respond: Thanks for your suggestion.

2. The gene sequences should be added in supplementary data.

Respond: Thanks for your suggestion. In the current version, the sequences and accession numbers of the eight candidate reference genes have been added in supplementary data (Table S1).

3. The gene names should be italicized in entire MS.

Respond: Thanks for your suggestion. We have checked the gene names throughout the MS.

4. The title needs correction. It should be..“ Evaluation of candidate reference genes for gene expression analysis in brassica leaf beetle, Phaedon brassicae (Coleoptera: Chrysomelidae)”.

Respond: Thanks for your suggestion.

5. line 117, “required” modified as “sufficient”.

Respond: Thanks for your suggestion. In the current version, this sentence has been modified to ‘our results proved that two references were sufficient for the reliable normalization in each condition’.

6. Line 219: delete the blank space after “of”.

Respond: Thanks for your correction.

7. Line 336: delete “that” to correct the sentence.

Respond: Thanks for your correction.

8. Use RT-qPCR instead of qRT-PCR throughout the whole ms.

Respond: Thanks for your correction. In the current version, this issue has been modified as you suggested.
