# Supplementary material for: Evaluation of candidate reference genes for gene expression analysis in the brassica leaf beetle, Phaedon brassicae (Coleoptera: Chrysomelidae)
Source: PLoS One. 2021 Jun 3;16(6):e0251920. doi: 10.1371/journal.pone.0251920 (PMC8174695; doi:10.1371/journal.pone.0251920)
Supplement: Attachment — Submitted filename: response.docx [file pone.0251920.s007.docx]

Dear editor and reviewers,

On behalf of my co-authors, we are grateful for giving us an opportunity to revise our manuscript, and we appreciate the anonymous reviewers for their positive and constructive comments on our manuscript. Based on these comments, we tried our best to polish the manuscript. The manuscript was modified systemically using the track change mode. The corrections are listed below point by point.

Review Comments to the Author

Reviewer #1: The authors made significant changes that improved the manuscript. I have a few minor changes below:

Line 63: do you mean 'LIFE' history.?
line 81: efficiency is misspelled
line 82: ...can AFFECT....
line 85: GENES
line 88: and TO eliminate...
line 96: delete 'speaking'
line 133: ....our results would facilitate future RESEARCH on ....
line 141: do you mean Petri dishes?
line 223: combination of reference GENES....
line 226: change 'OPERATED' by 'PERFORMED'
line 232: RANGING from ...
line 345: circumstances
line 374: howardi is part of the scientific name ...*Diabrotica undecimpunctata howardi*...
line 389: ..undecimpunctata howardi...
line 426: change 'give rise' to LEAD

Respond: Thanks for your constructive suggestion, and we have checked our writing thoroughly to prevent the misleading expression in the present version.

Reviewer #2: Authors did a great job to revise their manuscript. All questions raised have been answered. I don't have any further questions.

Reviewer #3: (No Response)
