# Supplementary material for: Evaluation of candidate reference genes for gene expression analysis in the brassica leaf beetle, Phaedon brassicae (Coleoptera: Chrysomelidae)
Source: PLoS One. 2021 Jun 3;16(6):e0251920. doi: 10.1371/journal.pone.0251920 (PMC8174695; doi:10.1371/journal.pone.0251920)
Supplement: Attachment — Submitted filename: response.docx [file pone.0251920.s008.docx]

Dear editor,

On behalf of my co-authors, we are grateful for giving us an opportunity to revise our manuscript, and we appreciate the anonymous reviewers for their positive and constructive comments on our manuscript. Based on these comments, we tried our best to polish the manuscript. An English language editing service editage was employed to polish the writing. The manuscript was modified systemically using the track change mode.

PONE-D-20-35478R2

Evaluation of candidate reference genes for gene expression analysis in the Brassica Leaf Beetle, Phaedon brassicae (Coleoptera: Chrysomelidae) PLOS ONE

Dear Dr. Zhang,

Thank you for submitting your manuscript to PLOS ONE. Unfortunately, the manuscript still needs extensive copy editing to rectify issues with sentence structure and verb tense. Frankly, in its present form the manuscript places too much of a burden on the reader to determine the authors' intended meaning for a number of sentences. I again suggest that you seek the input of a native English speaker or an English editing service.

We look forward to receiving your revised manuscript.

Kind regards,

J Joe Hull, Ph.D.
